# Treatment in Mental Hospitals

**Published:** 1914-02-07

**Authors:** Francis G. Scott

**Affiliations:** 71 Baker Street, W.


*To the Editor of* The Hospital.

### Treatment in Mental Hospitals.

### By Dr. Francis G. Scott.

*To the Editor of* The Hospital.

Sir,—It is more than remarkable how extremely backward the medical profession in England is in making use of psycho-therapeutic methods in the treatment of disease, especially when suggestion, both hypnotic and simple, has received the full sanction and recognition of the leading medical societies.

In the various medical schools and hospitals hypnotism is practically unknown, and the word psycho-therapy conveys little or no meaning to the average medical man.

The students of to-day are taught how to treat diseases of the brain, but of diseases of the mind they know little. The time, however, must come when the method of treatment will be adopted in our mental hospitals. For information on the subject we almost invariably have to turn to workers on the Continent or to translators of work. Such works as Dejerine and Gauckler's "Psycho-Neuroses and their Treatment by Psycho-Therapy" or Brill's "Psycho-Analysis" are only to be found in the medical libraries of a few of the London hospitals, and, needless to say, will not need rebinding for some considerable time.

No greater power than psycho-therapy lies in the hands of the practitioner, and no power is more neglected.

On the Continent numerous special "clinics" for its investigation are to be found, whilst in England there are only two conducted by medical men—one in Liverpool and the other in London—where psycho-therapeutic methods are systematically pursued.—Yours truly, Francis G. Scott.

